# Author Correction: Universal selective transfer printing via micro-vacuum force

**DOI:** 10.1038/s41467-023-44070-9

**Published:** 2023-12-06

**Authors:** Sang Hyun Park, Tae Jin Kim, Han Eol Lee, Boo Soo Ma, Myoung Song, Min Seo Kim, Jung Ho Shin, Seung Hyung Lee, Jae Hee Lee, Young Bin Kim, Ki Yun Nam, Hong-Jin Park, Taek-Soo Kim, Keon Jae Lee

**Affiliations:** 1 Department of Materials Science and Engineering, Korea Advanced Institute of Science and Technology (KAIST), 291 Daehak-ro, Yuseong-gu, Daejeon, 34141 Republic of Korea; 2 Division of Advanced Materials Engineering, Jeonbuk National University, 567 Baekje-daero, Deokjin-gu, Jeonju-si, Jeollabuk-do 54896 Republic of Korea; 3 Department of Mechanical Engineering, Korea Advanced Institute of Science and Technology (KAIST), 291 Daehak-ro, Yuseong-gu, Daejeon, 34141 Republic of Korea; 4 BSP Co., Ltd., 41-4, 170 Burim-ro, Dongan-gu, Anyang-si, Gyeonggi-do 14055 Republic of Korea

**Keywords:** Electrical and electronic engineering, Electronic devices

Correction to: *Nature Communications* 10.1038/s41467-023-43342-8, published online 26 November 2023

In this article the grant number RS-2023-00273231 was omitted. The original article has been corrected.

