# Supplementary material for: Universal selective transfer printing via micro-vacuum force
Source: Nat Commun. 2023 Nov 26;14:7744. doi: 10.1038/s41467-023-43342-8 (PMC10679119; doi:10.1038/s41467-023-43342-8)
Supplement: Supplementary file 1 — Supplementary Information [file 41467_2023_43342_MOESM1_ESM.pdf]

Universal Selective Transfer Printing via Micro-vacuum Force

Sang Hyun Park[1], Tae Jin Kim[1], Han Eol Lee[2], Boo Soo Ma[3], Myoung Song[3], Min Seo Kim[1], Jung Ho Shin[1], Seung Hyung Lee[1], Jae Hee Lee[1], Young Bin Kim[1], Ki Yun Nam[1], Hong-Jin Park[4], Taek-Soo Kim[3], and Keon Jae Lee[1]*

[1]S. H. Park, [+] T. J. Kim, [+] M. S. Kim, J. H. Shin, S. H. Lee, J. H. Lee, Y. B. Kim, K. Y. Nam, Prof. K. J. Lee
Department of Materials Science and Engineering, Korea Advanced Institute of Science and Technology (KAIST)
291 Daehak-ro, Yuseong-gu, Daejeon
34141, Republic of Korea
E-mail: keonlee@kaist.ac.kr

[2]H. E. Lee [+]
Division of Advanced Materials Engineering, Jeonbuk National University
567 Baekje-daero, Deokjin-gu, Jeonju-si, Jeollabuk-do
54896, Republic of Korea

[3]B. S. Ma, M. Song, Prof. T.-S. Kim
Department of Mechanical Engineering, Korea Advanced Institute of Science and Technology (KAIST)
291 Daehak-ro, Yuseong-gu, Daejeon
34141, Republic of Korea

[4]Dr. H.-J. Park
BSP Co., Ltd.
41-4, 170 Burim-ro, Dongan-gu, Anyang-si, Gyeonggi-do
14055, Republic of Korea

Keywords: selective transfer printing, micro-vacuum suction force, laser-induced etching, inorganic μLEDs, heterogeneous integration

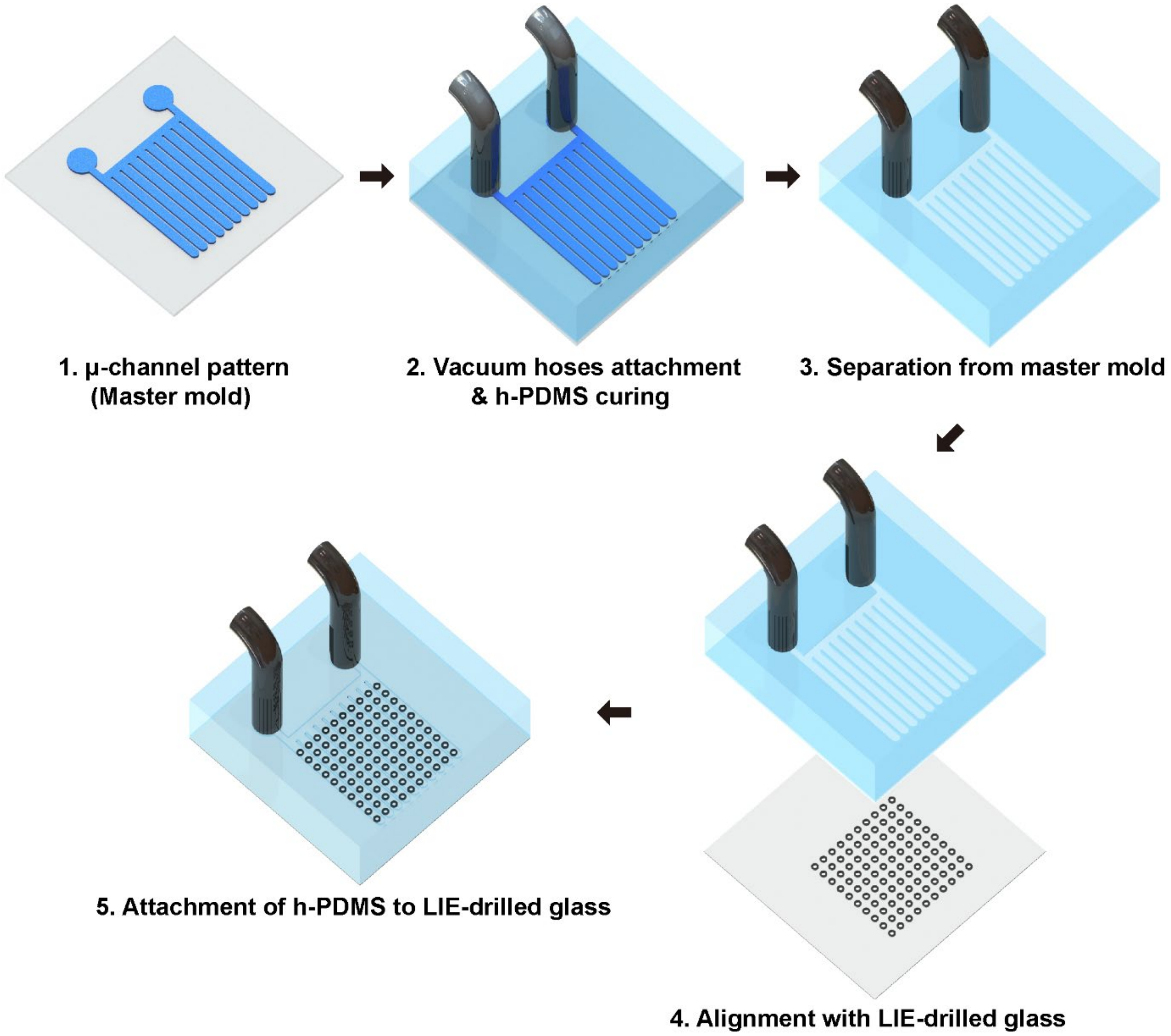

Fig. S1 | Fabrication process of VCM. The master mold is fabricated by μ-channel patterning on a glass substrate. The SU-8 100 photoresist is used for the μ-channel structure. After the attachment of the vacuum hoses to the master mold, the h-PDMS mixture (mass ratio of monomer to cross-linker is 1:1) is poured onto the master mold, followed by curing for 24 h at 75 °C. Afterward, the h-PDMS body is separated from the master mold and aligned with the LIE-drilled glass. The μ-pillar array is formed around the μ-hole array by the photolithography procedure. Finally, the VCM is fabricated by adhering the h-PDMS body to the LIE-drilled glass substrate.

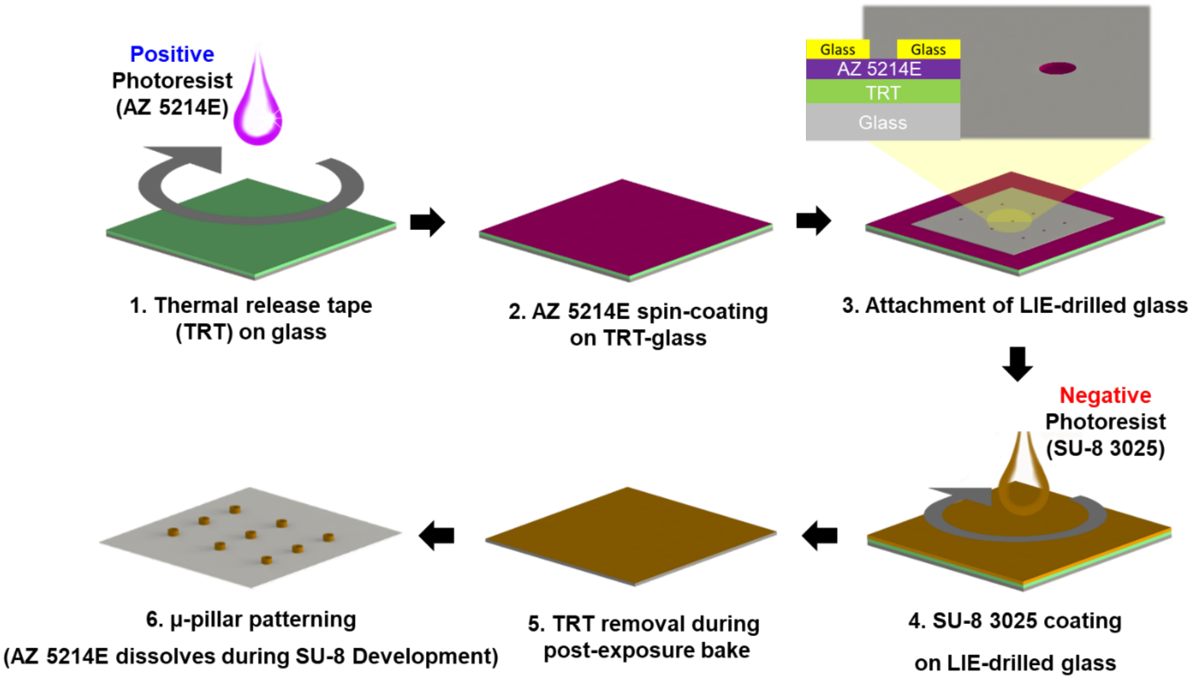

Fig. S2 | Fabrication process of μ-pillar. The thermal release tape (TRT) attached glass is utilized for the supporting substrate for μ-pillar fabrication. The AZ 5214E positive photoresist is spin-coated to adhere the LIE-drilled glass to the TRT-glass. In the curing process of AZ 5214E, the AZ 5214E fills inside the μ-hole for the flat surface during the following SU-8 patterning procedure. After the spin-coating of SU-8 3025 on the LIE-drilled glass, the supporting TRT-glass substrate is separated from the LIE-drilled glass by heat treatment at 105 °C. The heat treatment is performed for both TRT removal and SU-8 3025 baking process. Afterward, the μ-pillar is patterned by a photolithography procedure, followed by the removal of residual AZ 5214E inside the μ-pillar using acetone and isopropyl alcohol (IPA).

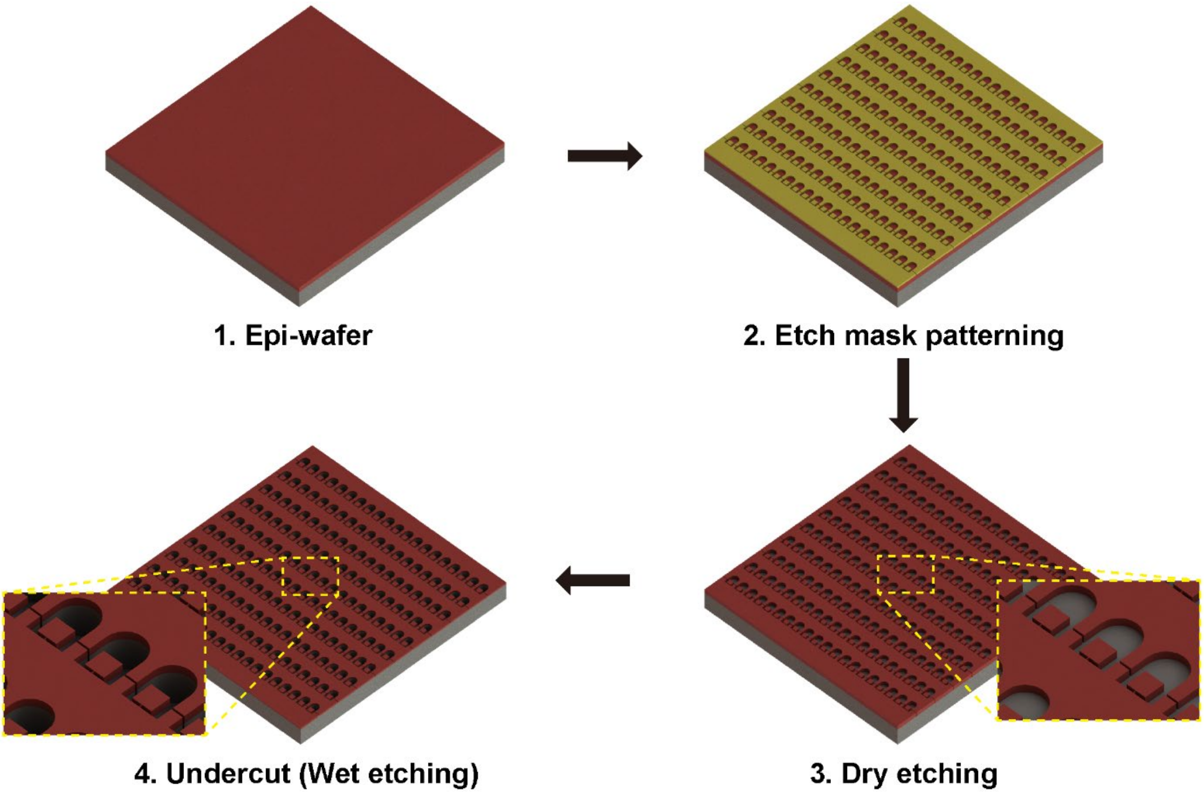

Fig. S3 | Fabrication process of freestanding microchip arrays. The AlGaInP-based epiwafer and SOG wafer are utilized for the fabrication of freestanding µLED and Si arrays, respectively. In the case of µLED arrays, the Cr/Au/Ni layer is patterned by a conventional photolithography process for the dry etch mask, followed by a dry etching procedure using Cl_2/Ar gas. After removal of etch mask layer, the GaAs substrate is selectively etched by hydrogen peroxide (H_2O_2) and citric acid ($C_6H_8O_7$)-based wet etchant for the freestanding structure of µLED arrays. The GaInP etch stop layer prevents the bottom of the AlGaInP active layer from damage during the undercut etching process. For the freestanding Si arrays, the negative resist (NR9-3000py) is patterned for the dry etch mask, followed by O_2/C_4F_8-based dry etching process to expose the underlying SiO_2 layer. Afterward, the SiO_2 layer, which originally supports the Si layer, is selectively removed by a wet etching process using BOE.

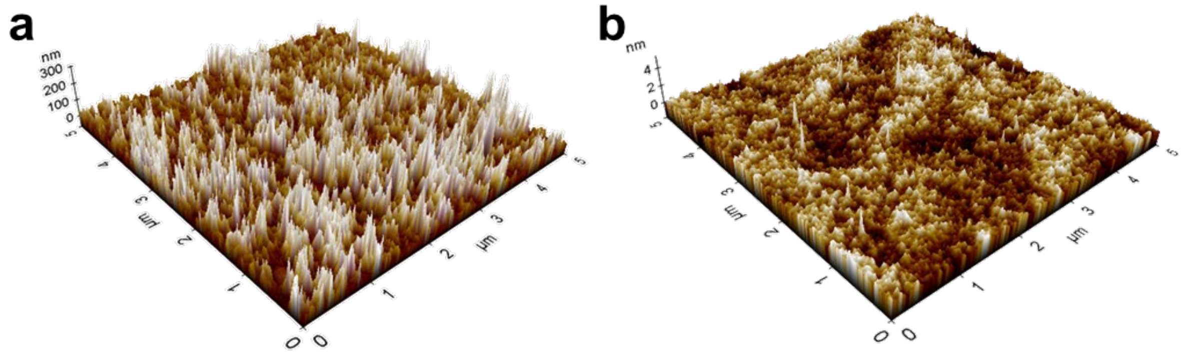

Fig. S4 | AFM results of a μLED and μ-pillar. a, Surface roughness of a μLED. The maximal roughness peak of d_{max} is 267.3 nm. **b,** Surface roughness of a SU-8 μ-pillar.

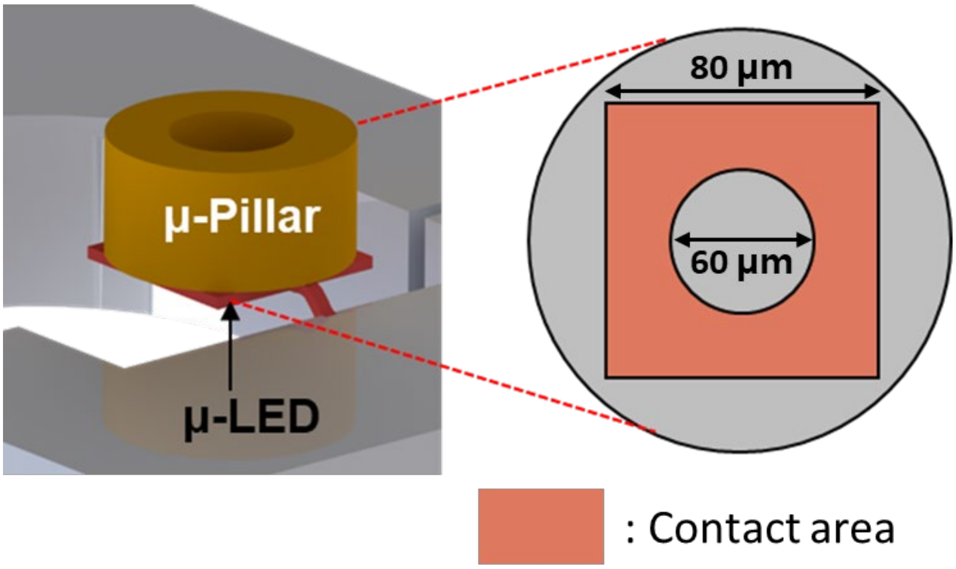

Fig. S5 | Contact area between a µLED and µ-pillar. Direct contact between an 80 µm-sized µLED and µ-pillar with a 60 µm inner radius during the µVAST process.

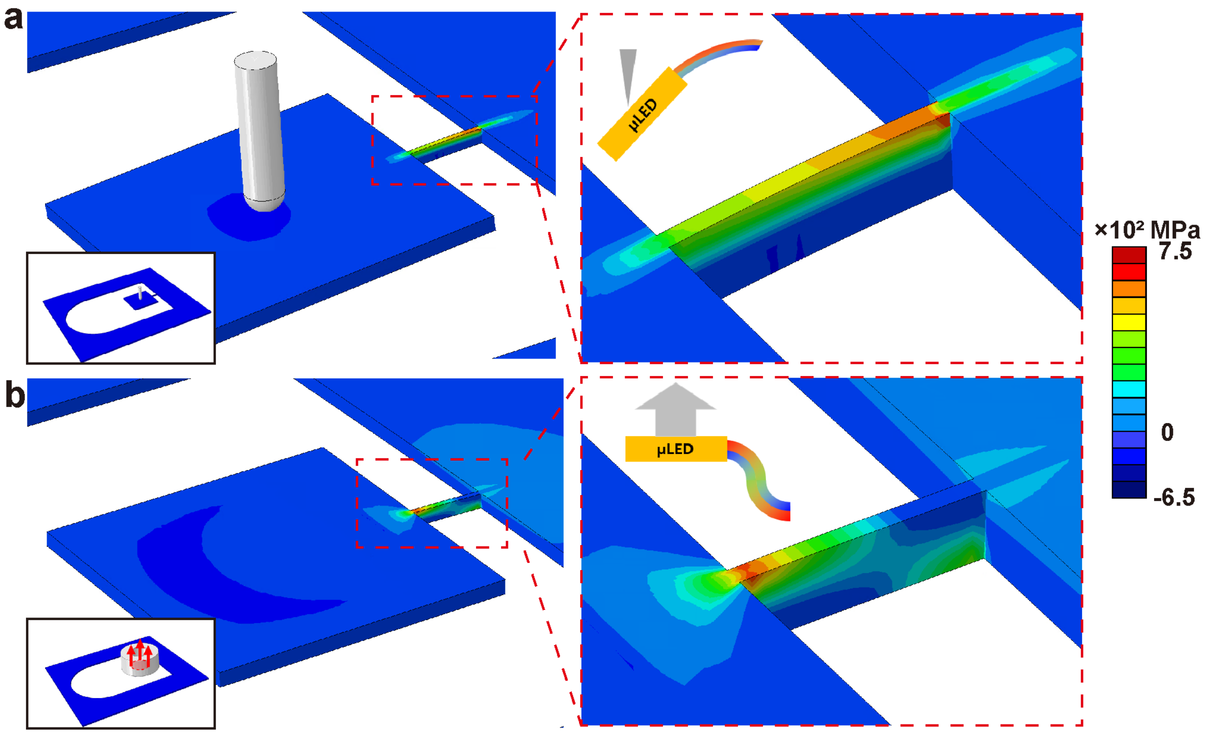

Fig. S6 | FEM simulation of stress distribution in µ-bridge. a, Stress distribution in µ-bridge during the nano-indentation. **b,** Stress distribution in µ-bridge during the pick-up process.

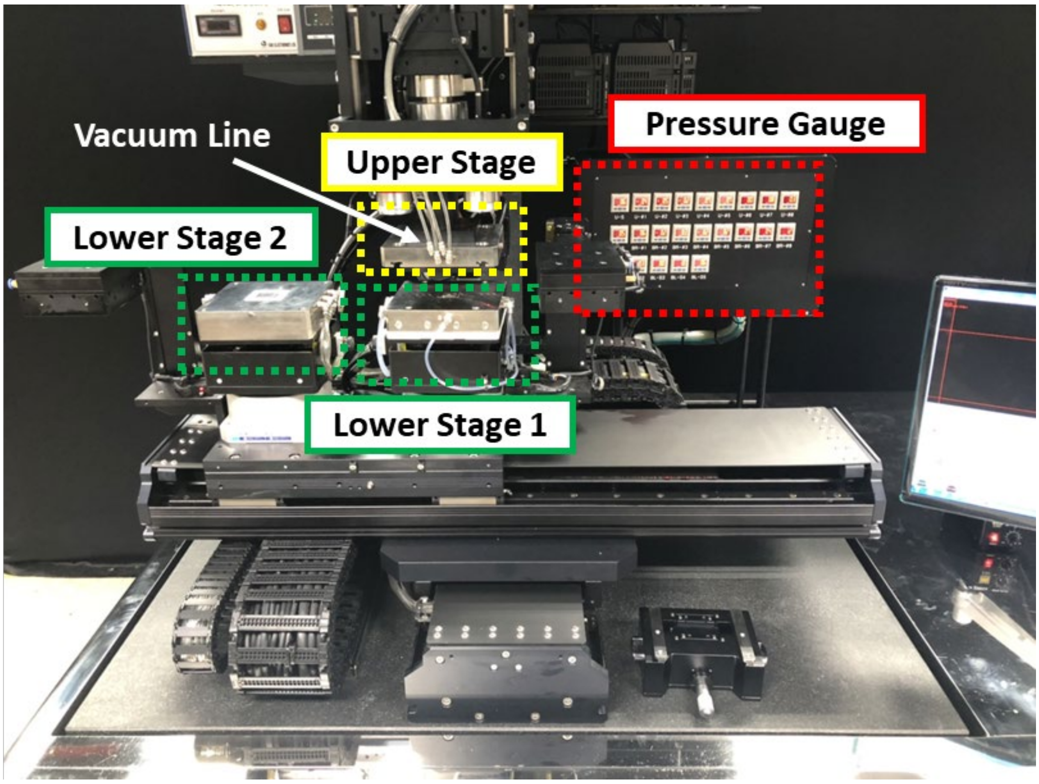

Fig. S7 | Transfer equipment for μVAST. The customized μVAST equipment consists of three stages, eight vacuum lines, three axes for stage moving, and three axes for stage tilting and rotation systems. The VCM is mounted on the upper stage, connected to the external vacuum pump through eight vacuum lines to independently control the micro-vacuum inside the μ-channels. The freestanding microchip arrays are loaded on the lower stages and aligned with the VCM through the alignment system. Two lower stages freely and precisely move in XYZ directions by stage moving axes for perfect alignment between μ-holes and microchips. Furthermore, the upper stage and lower stages keep parallel by three tilting and rotation axes for a conformal contact between the VCM and microchip arrays. Important parameters during the μVAST process such as the distance between stages and vacuum state are monitored in real-time by the laser displacement sensors and pressure gauges, respectively.

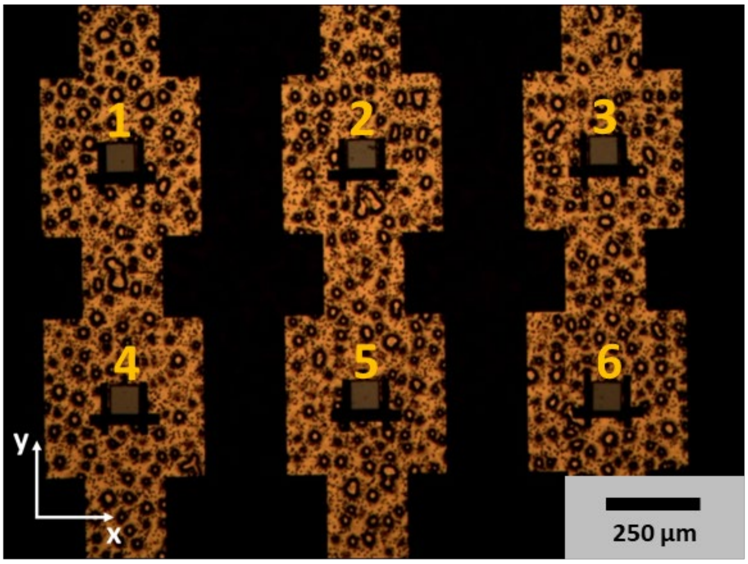

	#1	#2	#3	#4	#5	#6
X-axis	-5.935	-7.305	+7.761	+7.914	-6.696	-9.435
Y-axis	-2.511	-3.348	-4.337	-5.783	-3.652	-7.914

(unit: µm)

Fig. S8 | Positional error of transferred microchips via µVAST. The average positional inaccuracy of transferred µLEDs is 7.5 µm and 4.6 µm in the lateral and vertical directions, respectively.

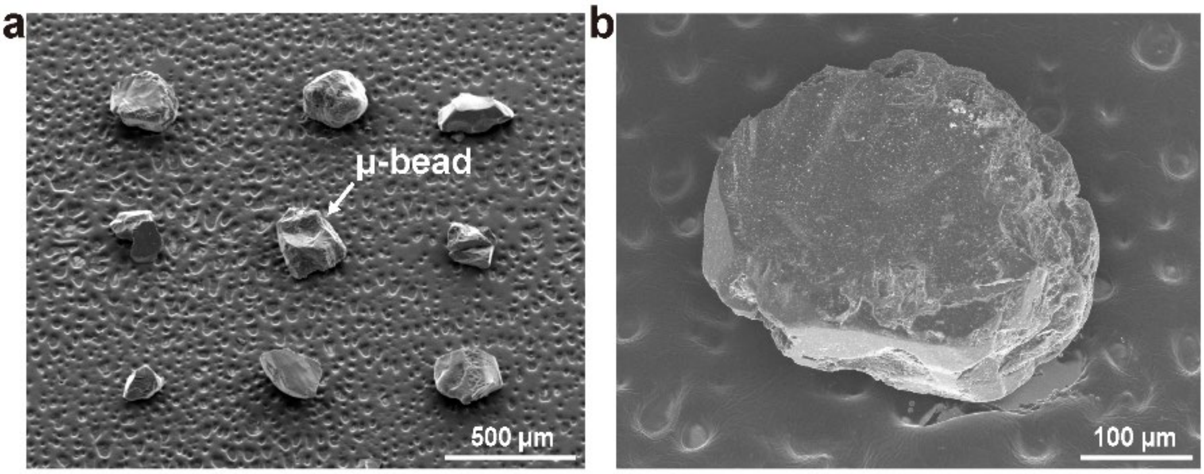

Fig. S9 | Transfer printing of non-planar objects via μVAST. a, SEM image of transfer-printed μ-bead arrays on a PI substrate. **b,** Magnified SEM image of a transferred μ-bead.

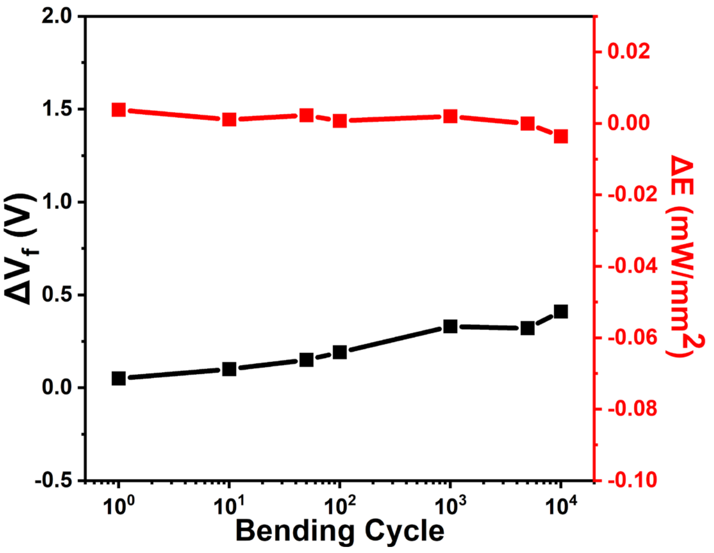

Fig. S10 | Durability test of the flexible μLEDs fabricated via μVAST. The mechanical durability of the flexible μLEDs is evaluated by periodic bending tests. The forward voltage and optical power density change within 0.41 V and 9 % after 10^4 bending cycles, respectively.

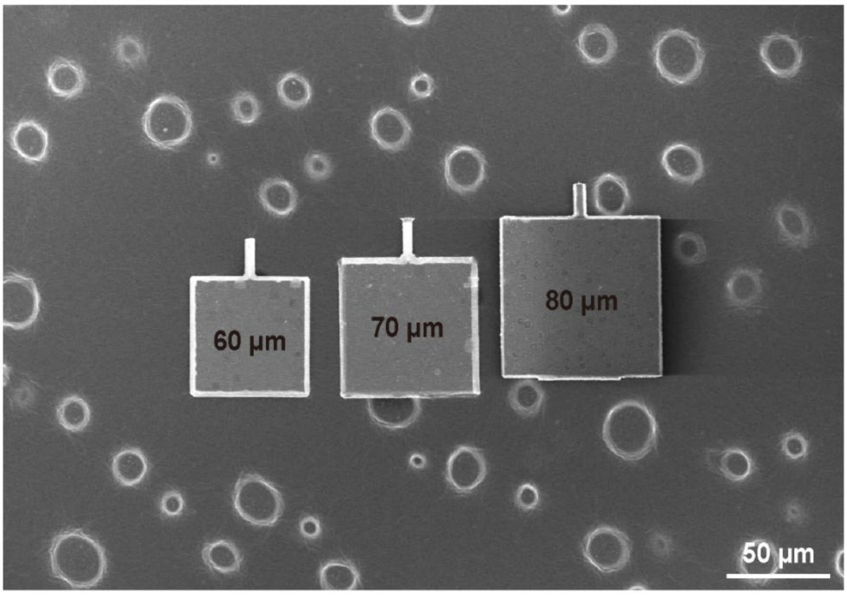

Fig. S11 | Transfer-printed microchips with various chip sizes. SEM image of 60/70/80 μm-sized microchips transferred on the flexible substrate. The minimum chip size of transferable microchips is 60 μm.

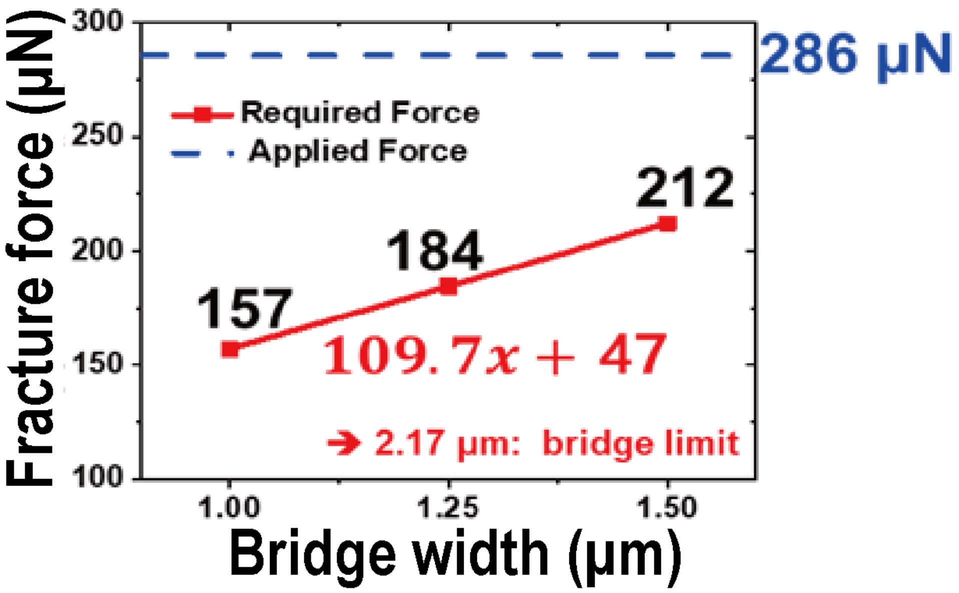

Fig. S12 | Maximum μ-bridge width for μVAST process. By extrapolating the relation between the fracture force and bridge width, the maximum bridge width is 2.17 μm for transfer printing via a micro-vacuum suction force of 286 μN.

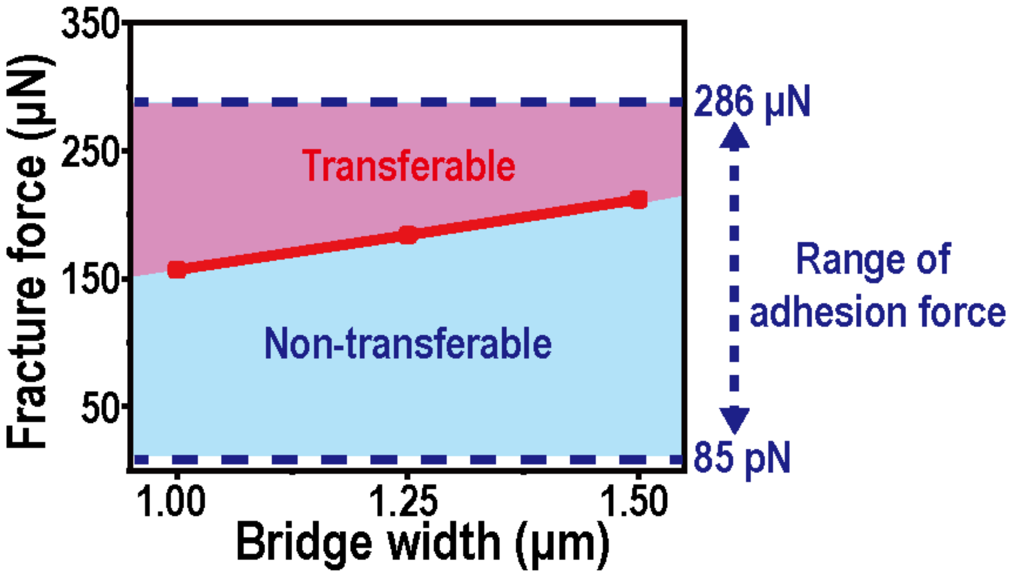

Fig. S13 | Range of adhesion force for μVAST. The adhesion force from 85 pN to 286 μN is applied on microchips by controlling the micro-vacuum suction force.

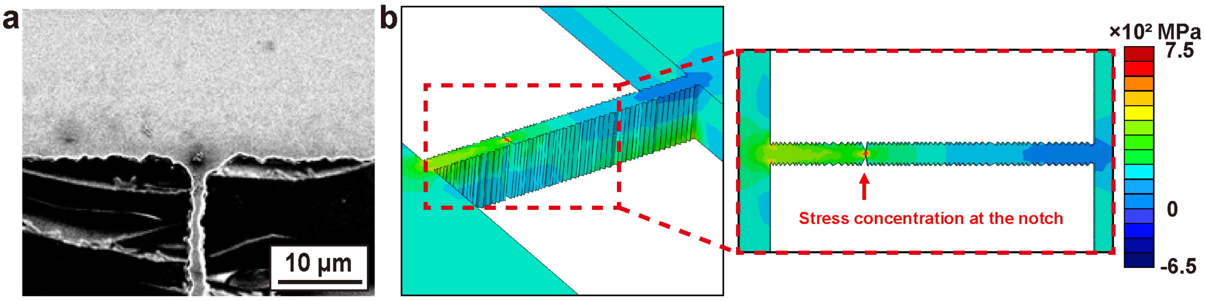

Fig. S14 | Fracture behavior in the serpentine μ-bridge. a, Magnified SEM image of the serpentine-shaped μ-bridge. **b,** FEM simulation of fracture behavior in serpentine-shaped μ-bridge.

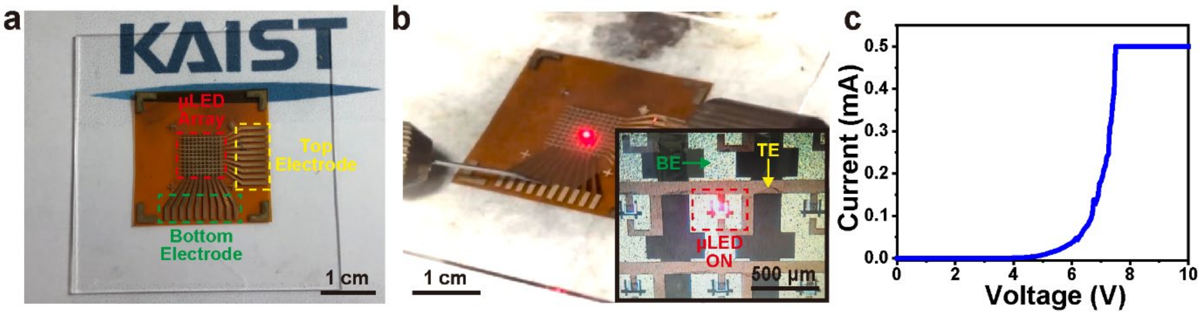

Fig. S15 | Individual driving of transferred μLEDs. a, Optical image of 10 × 10 flexible μLEDs with 10 bottom electrodes and 10 top electrodes. **b,** Optical image of an individually driven μLED. The inset shows the magnified OM image of an operating μLED. **c,** I-V characteristic of an individually driven μLED.

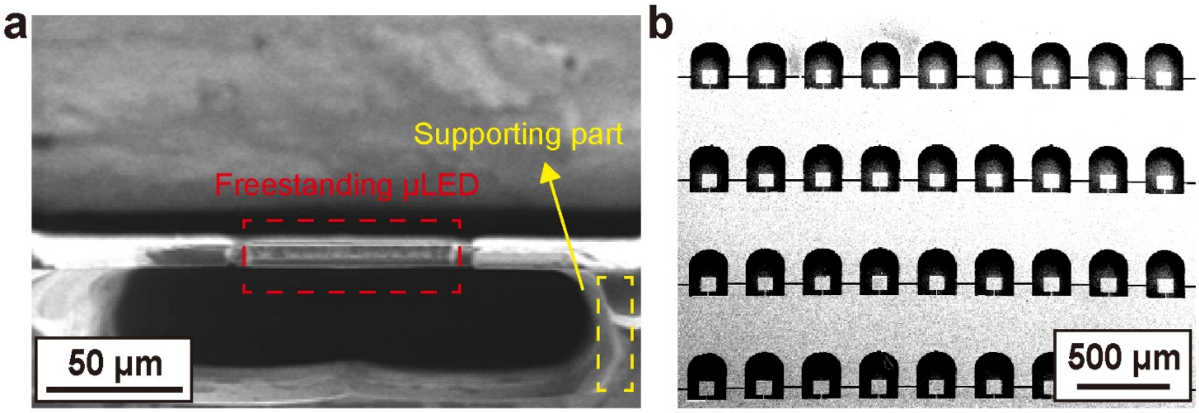

Fig. S16 | Factor related to the pitch of µLED arrays. a, Cross-section SEM image of the freestanding µLED. **b,** µLED arrays with a lateral pitch of 300 µm.

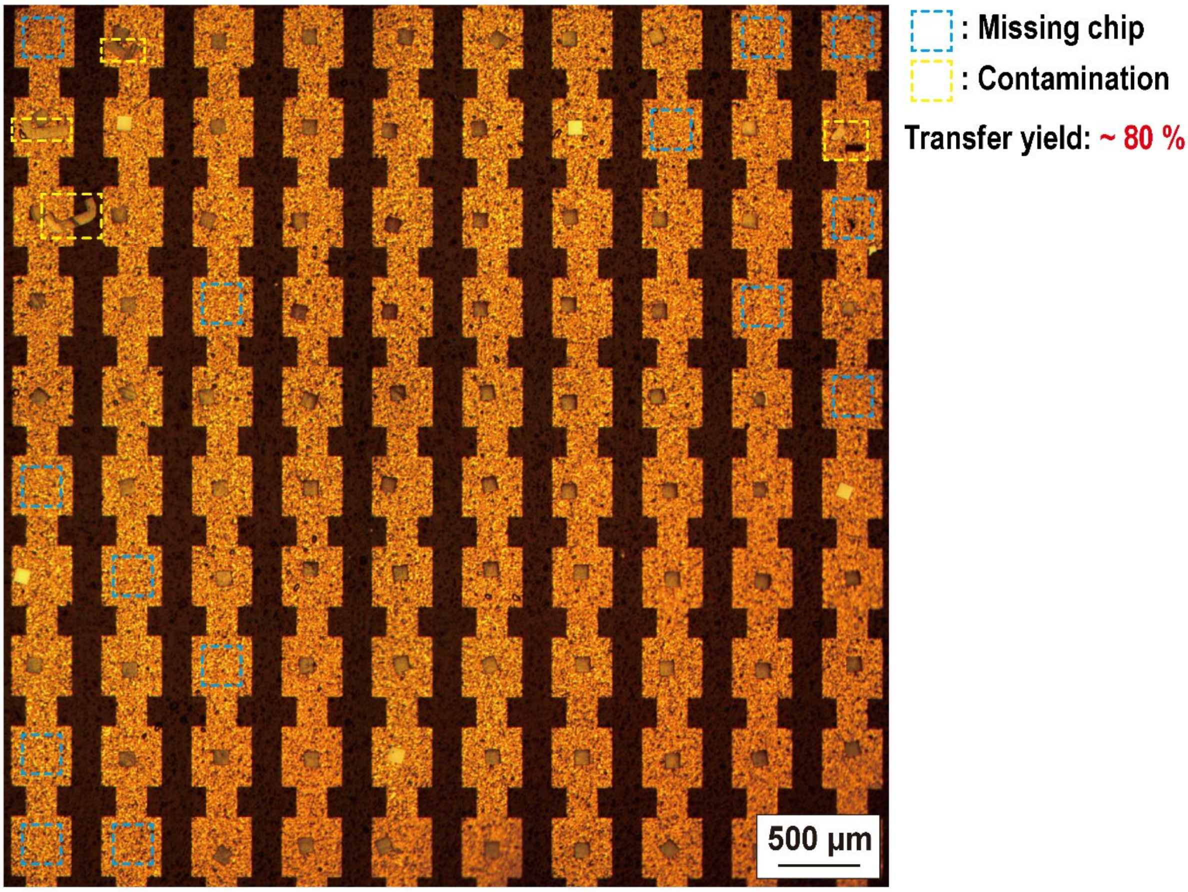

Fig. S17 | 10 × 10 µLEDs transferred via µVAST without µ-pillar arrays. The transfer yield of non-pillar µVAST is 80 % with 14 missing chips and 6 contaminations.

Supplementary Table 1 | Comparison between μVAST and conventional transfer printing technologies.

Transfer Method	Principle of Adhesion Control	Adhesion Switchability	Repeatability	Selectivity	Chip Damage	Large-area	Cost
Elastomeric transfer	Kinetic control (Peeling rate) of vdW force	<1000	Moderate	Moderate	Low	Moderate	Low
Electrostatic transfer	Electrostatic force	∞	Good	Moderate	High	Poor	High
Electromagnetic transfer	Electromagnetic force	∞	Good	Good	High	Poor	High
Laser-assisted transfer	Laser heating & Ablation	∞	Poor	Good	High	Good	High
Fluidic self-assembly	Gravity & Capillary force	-	Poor	Poor	Low	Good	Moderate
Pick-and-place	Vacuum suction force		Good	Good	Low	Poor	Moderate
**μVAST**	**Micro-vacuum suction force**	**3.364×10^6**	**High**	**High**	**Low**	**Good**	**Low**

Supplementary Table 2 | Comparison of transfer cost between μVAST and conventional transfer printing technologies.

Transfer process			μVAST	Pick-and-place	Elastomeric Transfer	Laser-assisted transfer	Electrostatic transfer
Transfer type			Mass transfer w/ selectivity	Individual transfer	Mass transfer w/o selectivity	Mass transfer w/ selectivity	Mass transfer w/ selectivity
Monthly production (ea/month)			10,000	1,000	4,000	10,000	10,000
Production cost ($/month)	μLED chips ($0.004/unit)		400,000	40,000	160,000	400,000	400,000
	Equipment depreciation (Life: 60 months)	Transfer equipment	4,000	4,000	4,000	8,000	8,000
		Other equipment	11,000	9,000	9,000	11,000	11,000
	Maintenance + Material costs ($/month)		50,000	37,500	44,000	50,000	50,000
**Cost per unit ($/unit)**			**46.5**	**90.5**	**54.25**	**46.9**	**46.9**

A rough cost for mass production of 4 × 4 cm^2–sized surface-lighting μLED patches (Lee et al., *Adv. Healthc. Mater.* **12**, 2201796, 2023) consisting of 100 × 100 μLED arrays using the μVAST process is calculated and compared to that of other transfer printing techniques. Transfer printing of 100 × 100 μLED arrays is carried out by four times selective transfer of 50 × 50 μLED arrays onto the 2 × 2 cm^2–sized flexible substrates. The costs of pre-transfer processes such as wafer manufacturing, μLED chip fabrication, and post-transfer procedures (packaging, and passivation) are assumed the same for all transfer technologies. The calculated production cost per month includes prices for the μLED chips, depreciation of equipment, maintenance, and material costs. The monthly production cost is closely related to the transfer processing speed: the faster the speed, the lower the cost. The equipment depreciation for transfer and other equipment is calculated by dividing the equipment prices by the expected equipment lifetime of 60 months. Maintenance and material costs contain the prices for factory space, labor, and raw materials.

Supplementary Note 1. Calculation of adhesion switchability in μVAST

The adhesion switchability of μVAST is the ratio of maximum adhesion force to minimum adhesion force, which is determined by the following equation (1).

$$\text{Adhesion switchability} = \frac{F_{max}}{F_{min}} = \frac{F_{suction}+F_{vdW}}{F_{vdW}} \qquad \textbf{(1)}$$

where F_{max} is the maximum adhesion force, which can be expressed as the sum of the micro-vacuum suction force and the van der Waals force. $F_{suction}$ is the vacuum suction force generated at the μ-holes and F_{vdW} is the van der Waals force between the microchips and the SU-8 μ-pillar. F_{min} is the minimum adhesion force, which can be defined by only the van der Waals force, because the vacuum suction force is removed by venting the μ-channel. F_{vdW}, the van der Waals interaction between two contact surfaces, could be calculated by equation (2).

$$F_{vdW} = \frac{A}{6\pi D^3} \times \text{Contact area} \qquad \textbf{(2)}$$

where A is the Hamaker constant and D is the separation distance between the microchips and the SU-8 μ-pillars[1]. Hamaker constant (A) is a physical coefficient to define the van der Waals force between two interacting bodies. Hamaker constant of A_{132} stands for the interaction between body *1* and *2* across medium *3*, which could be calculated by combining two known Hamaker constants[2].

$$A_{132} = \sqrt{A_{131}} \times \sqrt{A_{232}} \qquad \textbf{(3)}$$

Hamaker constant during μVAST can be expressed as $A_{Au\text{-}air\text{-}SU\text{-}8}$ because the Au thin-film is deposited on a freestanding μLED. The $A_{Au\text{-}air\text{-}SU\text{-}8}$ can be obtained by a combination of Hamaker constants as below[3–5].

$$A_{Au\text{-}air\text{-}Au} = 29 \times 10^{-20}\ \mathrm{J}$$

$$A_{SU\text{-}8\text{-}air\text{-}SU\text{-}8} = 2.53 \times 10^{-22}\ \mathrm{J}$$

$$A_{Au\text{-}air\text{-}SU\text{-}8} = \sqrt{A_{Au-air-Au}} \times \sqrt{A_{SU-8-air-SU-8}} \quad \textbf{(4)}$$

Separation distance (D) is measured by an atomic force microscopy (AFM) analysis because the separation distance of two contact surfaces is determined by the roughness of each surface[1]. According to the AFM results (Supplementary Fig. S4), a μLED shows a relatively large surface roughness value compared to that of the SU-8 μ-pillar. In this case, the distance between two rough surfaces is equivalent to the distance between a smooth surface and a rough surface, which is the maximal roughness peak of a rough surface. Based on this approximation, the separation distance between the μLED and the SU-8 μ-pillar is expressed as below.

$$D \approx d_{max} \text{ of μLED surface} \quad \textbf{(5)}$$

The contact area between the μLED and the SU-8 μ-pillar (Supplementary Fig. S5) can be calculated as follows:

$$\text{Contact area} = \text{Area of μLED} - \text{Inner circle area of μ-pillar} \quad \textbf{(6)}$$

Based on equations (2) ~ (5), the F_{vdW} of μVAST is 85 pN. As the $F_{suction}$ is 286 μN, derived from the air pressure difference between the inside and outside of the μ-channel during the μVAST process, the adhesion switchability of μVAST can be calculated as 3.364×10^6 by equation (1).

Supplementary References

1. Svetovoy, V. B. & Palasantzas, G. Influence of surface roughness on dispersion forces. *Adv. Colloid Interface Sci.* **216**, 1–19 (2015).

2. Visser, J. On Hamaker constants: A comparison between Hamaker constants and Lifshitz-Van der Waals constants. *Adv. Colloid Interface Sci.* **3**, 331-363 (1972).

3. Palasantzas, G., van Zwol, P. J. & de Hosson, J. T. M. Transition from Casimir to van der Waals force between macroscopic bodies. *Appl. Phys. Lett.* **93**, 121912 (2008).

4. Birleanu, C., Pustan, M., Voicu, R., Serdean, F. & Merie, V. Humidity influence on the adhesion of SU-8 polymer from MEMS applications. *MATEC Web of Conferences* **137**, 08002 (2017).

5. Leite, F. L., Bueno, C. C., da Róz, A. L., Ziemath, E. C. & Oliveira, O. N. Theoretical models for surface forces and adhesion and their measurement using atomic force microscopy. *Int. J. Mol. Sci.* **13**, 12773–12856 (2012).
