# Supplementary material for: Universal selective transfer printing via micro-vacuum force
Source: Nat Commun. 2023 Nov 26;14:7744. doi: 10.1038/s41467-023-43342-8 (PMC10679119; doi:10.1038/s41467-023-43342-8)
Supplement: Supplementary file 3 — Description of Additional Supplementary Files [file 41467_2023_43342_MOESM3_ESM.pdf]

<u>Description of Additional Supplementary Files</u>

Supplementary Movie 1: Entire process including LIE and µVAST,

Supplementary Movie 2: Pick-up and release procedures of µVAST
